# Challenges to Making the Decision to Stop Mass Drug Administration for Onchocerciasis: Lessons Learned from Nigeria

**DOI:** 10.4269/ajtmh.19-0951

**Published:** 2020-02-10

**Authors:** Darin S. Evans

**Affiliations:** USAID, Washington, DC

In this issue of the American Journal of Tropical Medicine and Hygiene, Frank Richards and others^1^ describe the decision to stop mass drug administration (MDA) with ivermectin for onchocerciasis after 25 years of treatment in two states in Central Nigeria. This decision is significant in that 1) at 2.2 million individuals, it represents the largest single population to ever eliminate transmission and stop MDA for onchocerciasis; 2) it is the first focus in Nigeria, the country with the largest at risk population in the world, to stop MDA; and 3) it is among the first countries to stop MDA using the updated 2016 WHO guidelines for stopping mass treatment.^2^ As such, the authors have noted many of the challenges in operationalizing these new guidelines and interpreting the results of their studies including analysis of historic baseline data and treatment decisions, assessing onchocerciasis transmission in posttreatment settings for lymphatic filariasis (LF) MDA, and capturing sufficient numbers of the black fly vector to make a stopping decision. These findings have implications for other countries in Africa that are approaching the decision to stop onchocerciasis MDA and highlight the need for further consensus and guidance moving forward.

Richards and others note that, while making the decision to stop MDA in districts historically defined as meso- and hyper-endemic, they needed to also consider neighboring districts that had not been treated under the original onchocerciasis program, but in which low-level transmission may have been measured at baseline in 1991. These districts were defined as “non-endemic” or “hypo-endemic,” terms that, in the early 1990s, were given to any district that was found to have a village-level microfilaria (Mf) prevalence of < 5%. The authors felt that it was important to include samples from districts where any transmission had been measured, and therefore included communities in these districts in their study. In Nigeria, these baseline designations were made using diagnostic tools and thresholds defined under the Onchocerciasis Control Program (1974–2002), whose principal focus was on vector control until ivermectin was introduced in 1987. Today, most of Africa has been designated under the African Program for Onchocerciasis Control (1995–2015), which focused principally on morbidity control and defined non-/hypo-endemic (i.e., “not” eligible for MDA) as < 20% nodule rate, roughly equivalent to 35–40% Mf, the prevalence less than which skin and eye disease are no longer expected. Although this threshold was eventually reduced to 10% Mf and later 5% Mf between 2009 and 2015, updates to historic nodule rates and expansion of MDA into these areas has been sporadic, and the true transmission status remains unknown in many areas. Consideration of these areas and decisions on whether to include them in future assessments or to start ivermectin MDA need to be made as countries begin to take stock of their transmission foci.

One influential factor in making decisions to assess or treat non-/hypo-endemic areas will be the history of LF treatment. The authors note that LF MDA (a combination of the same drug, ivermectin, plus albendazole) began at scale throughout the two Nigerian states under evaluation in 2003 and lasted 8–11 years. What the impact of those treatments may have been on the non-/hypo-endemic onchocerciasis communities is not clear. Entomologic surveys conducted for onchocerciasis in some of these communities during this study did not show any positive flies, and the authors concluded that either transmission in these sites never existed or that 8–11 years of LF MDA had broken onchocerciasis transmission. How other countries are to assess similar areas remains to be seen, but the scope of this challenge may be significant. Recent estimates have suggested that as many as 96 million people live in areas that are co-endemic for both LF and onchocerciasis.^3^ Data from the WHO have shown that there are as many as 2,577 districts where the status of onchocerciasis transmission is either unknown or incomplete.^4^ Of these, at least 713 are in districts receiving MDA for LF. At present, there are no formal strategies for assessing the status of onchocerciasis transmission in the context of LF MDA. Countries could choose to conduct full stop-MDA surveys in each of these districts, but such surveys are large and costly and would require conducting them in districts where transmission has likely never existed. How endemic countries will deal with similar districts in determining both when to stop LF MDA and when to continue with onchocerciasis MDA must be clarified. One opportunity might be in linking an onchocerciasis assessment with LF transmission assessment surveys which are required of LF programs to make their own stopping decisions. Such an integrated approach could reduce costs while simultaneously making programmatic decisions for both onchocerciasis and LF.

Another area highlighted by the authors was assessment of the black fly vector. A previous study from a subset of the sites included in this study noted the challenge of collecting a sufficient number of black flies using human landing capture (HLC).^5^ The present study made use of the Esperanza window trap, which can allow capture of up to five times the number of flies as an individual HLC.^6^ However, the trap eliminates the ability to determine a biting rate, which in turn eliminates the ability to calculate the annual transmission potential, a key indicator of transmission in areas with low vector density. If such tools are to be used, developing other indicators, such as a landing rate as a proxy for biting rate, may be necessary.

In 2007, this journal published one of the first articles to show the elimination of onchocerciasis transmission using the 2001 WHO guidelines for stopping MDA, in Santa Rosa, Guatemala.^7^ Many of the operational practices outlined in that article were later taken up to make stopping decisions elsewhere in the Americas, as well as in African countries including Uganda and Sudan. Today, four countries in the Americas have been verified free of onchocerciasis, and 20 foci across both the Americas and Africa have been able to stop MDA using the lessons learned from that study. The WHO has set a target of 10 countries verified free from onchocerciasis and at least 34 foci to have stopped MDA by 2030. Over the next decade, as the number of onchocerciasis surveys increases, the lessons learned in Nigeria will help to inform other countries in their upcoming decisions. This will, however, require consensus and a unified approach to interpreting historic data and treatment decisions, clarification of how to identify evidence of onchocerciasis from districts treated for LF, and improving tools for entomology and programmatic decisions.
